# The World of Pseudogenes: New Diagnostic and Therapeutic Targets in Cancers or Still Mystery Molecules?

**DOI:** 10.3390/life11121354

**Published:** 2021-12-07

**Authors:** Maciej Stasiak, Tomasz Kolenda, Joanna Kozłowska-Masłoń, Joanna Sobocińska, Paulina Poter, Kacper Guglas, Anna Paszkowska, Renata Bliźniak, Anna Teresiak, Urszula Kazimierczak, Katarzyna Lamperska

**Affiliations:** 1 Greater Poland Cancer Centre, Laboratory of Cancer Genetics, Garbary 15, 61-866 Poznan, Poland; maciej.stasiak96@gmail.com (M.S.); kozlowskaj97@gmail.com (J.K.-M.); a.s.sobocinska@gmail.com (J.S.); kacper.guglas@gmail.com (K.G.); apaszek00@gmail.com (A.P.); renata.blizniak@wco.pl (R.B.); anna.teresiak@wco.pl (A.T.); 2 Greater Poland Cancer Centre, Research and Implementation Unit, Garbary 15, 61-866 Poznan, Poland; paulina.poter@gmail.com; 3 Faculty of Biology, Institute of Human Biology and Evolution, Adam Mickiewicz University, Uniwersytetu Poznańskiego 6, 61-614 Poznań, Poland; 4 Greater Poland Cancer Center, Department of Oncologic Pathology and Prophylaxis, Poznan University of Medical Sciences, Garbary 15, 61-866 Poznan, Poland; 5 Department of Pathology, Pomeranian Medical University, Rybacka 1, 70-204 Szczecin, Poland; 6 Postgraduate School of Molecular Medicine, Medical University of Warsaw, 61 Zwirki and Wigury, 02-091 Warsaw, Poland; 7 Faculty of Biology, Adam Mickiewicz University, Umultowska 89, 61-614 Poznan, Poland; 8 Department of Cancer Immunology, Medical Biotechnology, Poznan University of Medical Sciences, 8 Rokietnicka Street, 60-806 Poznan, Poland; ukazimierczak@ump.edu.pl

**Keywords:** pseudogenes, lncRNA, non-coding RNA, ceRNA, transcription regulation, cancer, biomarker, liquid biopsy, TCGA

## Abstract

Pseudogenes were once considered as “junk DNA”, due to loss of their functions as a result of the accumulation of mutations, such as frameshift and presence of premature stop-codons and relocation of genes to inactive heterochromatin regions of the genome. Pseudogenes are divided into two large groups, processed and unprocessed, according to their primary structure and origin. Only 10% of all pseudogenes are transcribed into RNAs and participate in the regulation of parental gene expression at both transcriptional and translational levels through senseRNA (sRNA) and antisense RNA (asRNA). In this review, about 150 pseudogenes in the different types of cancers were analyzed. Part of these pseudogenes seem to be useful in molecular diagnostics and can be detected in various types of biological material including tissue as well as biological fluids (liquid biopsy) using different detection methods. The number of pseudogenes, as well as their function in the human genome, is still unknown. However, thanks to the development of various technologies and bioinformatic tools, it was revealed so far that pseudogenes are involved in the development and progression of certain diseases, especially in cancer.

## 1. Pseudogene Transcripts

The pseudogene is a copy of a gene that has lost its original function due to the accumulation of mutations, such as frameshift and the presence of premature stop-codons and relocation of genes to inactive heterochromatin regions of the genome [1]. The first study about these molecules was performed by Jacq et al. when they reported the existence of a group of untranscribed genomic sequences homologous to the 5S DNA in *Xenopus laevis* [2]. After that, pseudogenes have been identified to be widely present in the genomes of most organisms, ranging from prokaryotes to eukaryotes [3,4]. At first, they were branded as non-coding, “junk DNA”. However, experimental data obtained during recent years indicate that 10% of approximately 16,000 identified pseudogenes are transcribed, and roughly 19% of known human lncRNAs are the products of pseudogene transcription [5,6,7]. Pseudogenes are divided into two large groups according to their primary structure and origin: processed and unprocessed. The first ones are formed by integration into new genome sites of cDNAs produced by the reverse transcription of parental genes. Due to this reason, processed pseudogenes do not contain introns. The majority of these molecules have a poly(A) sequence at the 3′end due to the mRNA 3′end polyadenylation process. In addition, such pseudogenes are flanked by duplicated integration sites 5 to 20 bp in length. Dong et al. identified a subgroup of processed pseudogenes that are a result of circ-RNA transcription. Such pseudogenes usually lack the 3′end poly(A) sequences. Moreover, they feature the reverse order of introns as compared to the original mRNAs [8].

**Figure 1:**
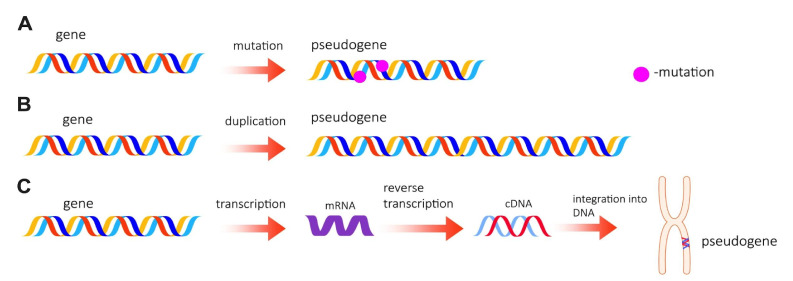
The origin of the pseudogenes in the genome. Pseudogenes arise as a result of changes in the parental gene due to mutations (**A**), duplications in DNA (**B**), or changes in the transcription process and integration of a reversed transcribed product into the genome (**C**).

The second group of pseudogenes, in comparison to processed pseudogenes, contain in their sequence introns and can be unitary (orphan) or duplicated. Unitary pseudogenes are derived from single-copy functional genes, which accumulated spontaneous mutations during evolution and have lost their primary functions. Therefore, unitary pseudogenes have no paralogs in the same genome but may have orthologs in the relative species [9]. Duplicated pseudogenes arise from tandem duplications of genes during an unequal crossing-over process. The duplicated gene can undergo further mutations, which convert it into a completely new pseudogene. Because of the mechanism of origin, duplicated pseudogenes are situated on the same chromosomes as their parental genes [10]. The origin of the pseudogenes in the genome is shown in Figure 1.

## 2. Pseudogene Functions

**Figure 2:**
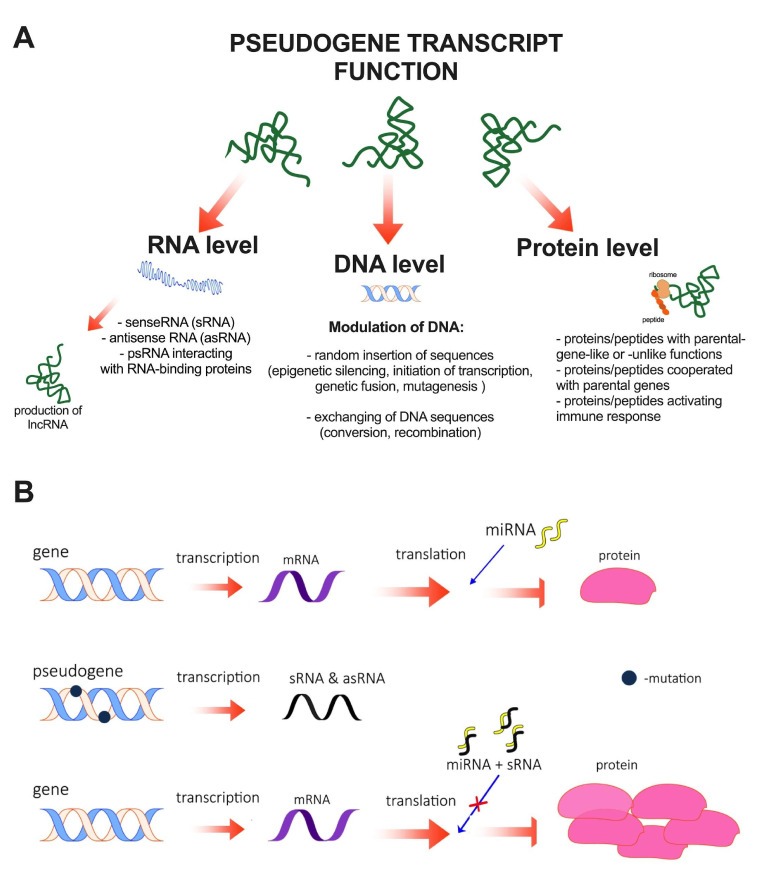
The regulatory function of the pseudogenes. Pseudogene interactions on different molecular levels include RNA, DNA, and protein molecules (**A**). Regulation of parental gene transcripts by pseudogene is possible by using the molecular sponge mechanism. The pseudogene transcripts possessing the same miRNA binding sites as parental gene capture miRNAs from the cellular environments which are not able to inhibit the transcript and specified protein is translated (**B**).

Pseudogene transcripts were thought to be non-functional transcription noise. One of the probable reasons for this perception of pseudogene functions was the assumption that these regions are in principle non-functional, which meant that they were not studied in this regard [11]. However, as is often the case in science, random results or the insight of researchers have led to more and more data pointing to the functionality of pseudogenes. It is known that some pseudogenes take part in many different important biological processes such as immunological response, catalytic reactions, signaling pathway regulations, in the process of architecture changes of chromatin or genome, and functions as transcription and translation factors, elements of gene conversion, dimerization factors, stabilizing elements, or structural proteins [11]. All of these underline that pseudogenes are important elements of the genome regulatory network. We now know that pseudogenes perform their functions at different levels, which include interaction at the RNA, DNA, and protein levels. The schematic illustration of pseudogenes regulatory function is shown in Figure 2A.

The first functional level is interaction and regulation of RNAs molecules. As mentioned earlier, 10% of all pseudogenes are transcribed into RNAs (psRNAs), and that RNAs participate in the regulation of parental gene expression at both transcriptional and translational levels through senseRNA (sRNA) and antisense RNA (asRNA). sRNA regulates the expression of their parental gene mRNA through competition for miRNA. Due to the significant similarity, they share miRNA binding sites, whose binding to miRNAs ensures the regulatory functions of these RNA molecules in both the nucleus and the cytoplasm [12]. The higher the pseudogene transcription activity, the higher the number of miRNA molecules that bind to its sRNA, which depletes their intracellular pool and reduces suppression of the parental gene expression [13].

psRNAs can compete for the binding not only of miRNAs but also various regulatory proteins and protein complexes, including RNA-binding proteins and transcription factors. In this case, psRNAs can act as decoys. For example, reduced expression of the high mobility group A1 protein (*HMGA1*) associated with type 2 diabetes may be caused by upregulated transcription of the *HMGA1p* pseudogene, which competes with the 3′UTR of *HMGA1* gene for the protein factor αCP1 critical for the stability of its mRNA [14].

asRNAs are involved in many regulatory mechanisms of their parental genes, Figure 2B. For example, asRNAs can form duplexes with their parental gene sRNAs, which may give rise to siRNAs [15,16,17]. Recently, asRNAs were found to interact with PIWI proteins (piRNA) in animal spermatozoa and germline cells [18,19]. The main function of typical piRNAs is inhibition of transposon activity in germline cells, e.g., at the transcription level, by heterochromatinization of the corresponding genetic loci through methylation of DNA or histones [19]. asRNAs can also enhance the transcription process, e.g., one of six expressed pseudogenes of *POU5F1, OCT4pg5,* generates asRNA that transports histone methyltransferase to the *POU5F1* gene promoter. This process is accompanied by trimethylation of histone H3 Lys27 on the chromatin surrounding the promoter and inhibition of the gene transcription [20]. While *POUF5F1* has several pseudogenes, *PTENP1* pseudogene can be universal. *PTENP1* has three transcripts: one sRNA and two overlapping asRNA isoforms, α and β. Isoform α causes heterochromatinization and repression of *PTEN* gene promoter, sRNA competes with PTEN mRNA for miRNA, i.e., represents typical ceRNA and positive gene regulator function and isoform β stabilizes sRNA via interaction of its 3′ end with the 5′ end sequence of the sRNA [21].

Another function of pseudogenes is production of long non-coding RNAs (lncRNAs). These transcripts are long non-coding RNA molecules without protein products but in some cases, short peptides are generated. lncRNAs function as regulators of transcription by activation of specific genes, modulators of protein factors and chromatin, guides for specific ribonucleoprotein complexes as well as scaffolds for specified ribonucleoproteins [22]. It is also postulated that lncRNAs function as molecular sponges for miRNA, e.g., *ZFAS1* lncRNA, which regulates *miR-150-5p* in HNSCC [23]. lncRNAs could probably be used as biomarkers in oncology, but the role of some of these transcripts is not fully understood [22,24,25,26,27]. Detailed information about lncRNAs is described by us elsewhere [24,28].

It should be emphasized that some evidence is in opposition about the function of pseudogenes as the elements of the ceRNA network and it is postulated that they are true but at unphysiological levels [29,30].

The second type of regulation is the ability to modulate DNA, which is manifested by random insertion of a pseudogene sequence into the parental or other host gene as well as causing DNA sequence exchange between the pseudogene and parental gene [31]. The insertion of pseudogene sequence can cause different biological effects: (i) epigenetic silencing, (ii) initiation of transcription, (iii) genetic fusion, or even (vi) mutagenesis. These modifications induce changes in expression level of specific genes or cause alternative functions of them, which could induce carcinogenesis [32,33,34,35]. Another possibility is exchanging DNA sequences between the pseudogene and parental gene. In this case, the conversion as well as recombination is possible [36,37]. One of the examples of this is the rearrangements between the *BRCA1* gene and *BRCA1* pseudogene that causes origin of mutated alleles, which lack promoter, are changes in the exons and lack the initiation codon [37]. Exchanging DNA sequences between pseudogene and parental gene strongly influences the genome and could lead to inactivation of suppressor genes or activation of oncogenes [36,37].

The last pseudogene function is the possibility of influencing the genome and transcriptome by protein or peptide. Paradoxically, some pseudogenes such as some lncRNAs have open reading frames and encode proteins or peptides and these products could play a regulative function in a cell. These pseudo-proteins or -peptides could have parental gene-like or -unlike functions, cooperate with parental genes or even activate immune response [31]. One of the examples is *PGAM3* pseudogene with protein product with unknown function in humans and classified as processed pseudogene. Another example is *OCT4* pseudogenes, which are highly similar to *OCT4* gene [38]. Recent studies indicated that the OCT4pg1 protein is involved in changes in cancer phenotype in triple-negative breast cancers by activation of the *Notch* pathway [39]. Suo et al. observed that *OCT4* pseudogenes, *Oct4pg5* and *Oct4pg1*, are transcribed in cancer and regulates the *OCT4*. Moreover, these pseudogenes probably generated artifactual results about *OCT4* [38]. Similar results obtained by Zhao et al. demonstrated that *OCT4* pseudogenes, *OCT4pg1*, *OCT4pg3* and *OCT4pg4*, are transcribed and translated in glioma and breast without OCT4 products [39]. These observations underline the need for further examination and verification of some results and define the role of pseudogenes’ proteins. To make it even more interesting, some pseudogenes code not proteins similar to the parent genes, but their truncated forms in the form of peptides. *BRAF* pseudogene 1 (*BRAFP1*) has many stop codons and shortened peptides are generated in contrast to translated protein from BRAF gene. Pseudo-BRAF peptide was described in the context of thyroid cancer and activates the *MAP* kinase signaling pathway, leading to tumorigenesis. Moreover, it was indicated that *BRAF* pseudogene 1 transcripts were negatively correlated with *BRAF* mutation [40]. However, other studies indicated that *BRAFP1* functions as a competitive endogenous RNA [41]. The last example is the antigen-like function of pseudo-proteins/peptides which possesses the capability of simulation of the immune system. Moreau-Aubrey et al. indicated that the processed pseudogene *NA88-A* codes for a new antigen recognized by a CD8(+) T cell clone on melanoma. Interestingly, the *NA88-A* parental gene, *HPX42B*, codes for hemoprotein and is transcribed in a variety of normal tissues [42].

All of these examples clearly show that pseudogenes are functional molecules which were missed in investigations or naturally deeply hidden in the wide network of cellular interactions between DNA, RNA and protein molecules.

## 3. Involvement of Pseudogenes in Cancers

Thanks to the incredible development of next-generation sequencing technology and bioinformatics tools, a large number of pseudogenes have gradually been discovered. As mentioned earlier, pseudogenes can interact in various ways with DNA, RNA, and proteins participating in the modulation of target gene expression, particularly their parental genes. Therefore, these molecules are involved in the development, and progression of certain diseases, especially cancer [43]. Although comprehensive pseudogene studies have just been started, they revealed the broad participation of pseudogenes in cancer development and diagnostics.

**Figure 3:**
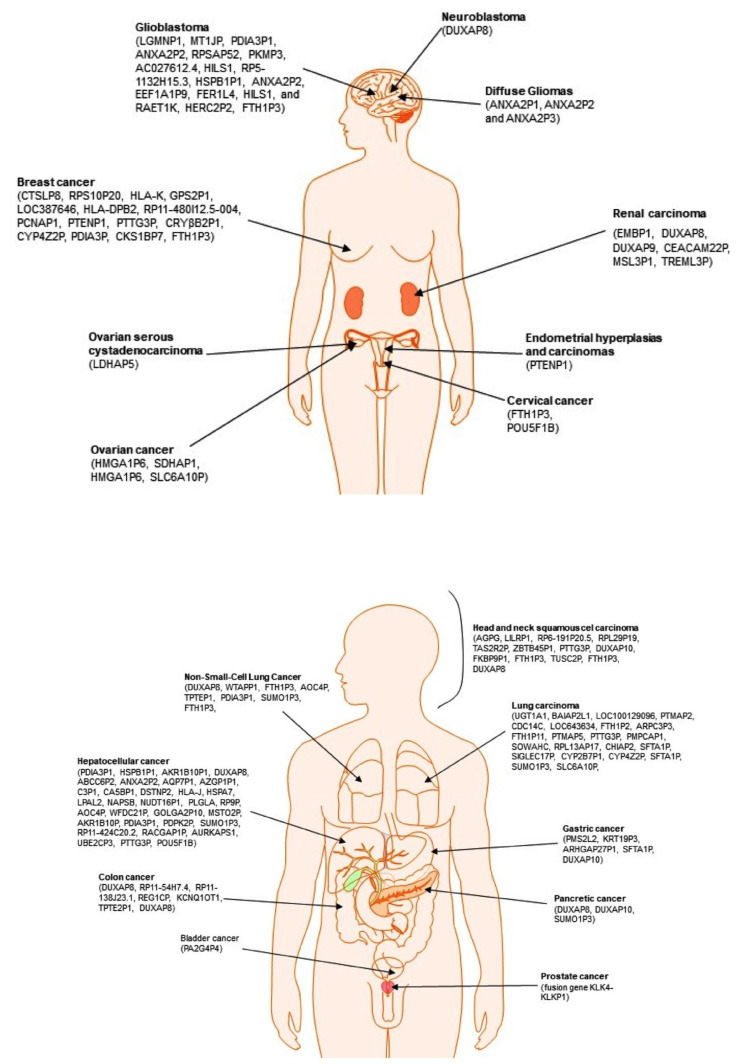
The pseudogenes identified as diagnostic, prognostic, or predictive biomarkers in human cancers.

Based on available literature data and public databases, selected pseudogenes can be classified as the predictor, inheritance, or prognostic biomarkers. Chosen pseudogenes whose expressions are noticeably changed in the group of cancers located in the abdomen and bones, chest, and head and neck area are presented in Figure 3.

### 3.1. Cancers Located in the Abdomen and Bones

In the abdomen and bones area, 73 pseudogenes in such cancers as bladder carcinoma, cervical carcinoma, colorectal cancer, osteosarcoma, and more, in tissue, plasma, blood, and urine samples have been indicated. In the tissues of acute myeloid leukemia patients, *BMI1P1A*, *OCT4*, and *POU5F1B* are three gene signatures that divide individuals into high-risk and low-risk groups [44]. *PA2G4P4* is overexpressed in bladder cancer patient tissues and cell lines [45]. *GBP1P1* and *PTTG3P* were observed in microarray analysis and validated by qRT-PCR in tissues of cervical carcinoma [46]. *FTH1P3* and *POU5F1B* are upregulated in cervical cancer patient samples and cell lines [47,48]. In colon cancer tissues, *DUXAP8*, *RP11-54H7.4*, and *RP11-138J23.1* show elevated expression in advanced tumor stages [49]. In colorectal cancer tissues, increased *KCNQ1OT1* (as well as *PNN*) is associated with shorter DFS of individuals in stage III treated with 5-FU adjuvant therapy [50]. *REG1CP*, *TPTE2P1*, and *DUXAP8* are upregulated in colorectal cancer patient samples and cell lines [51,52,53].

In tissue and blood samples from endometrial hyperplasia and carcinomas patients, *PTENP1* was methylated in all analyzed tissues, except for the peripheral blood. No differences were determined between the EC and EH groups [54]. In gastric adenocarcinoma patient tissues, *PMS2L2* and *SFTA1P* were found to be downregulated [55,56]. Additionally, three pseudogenes, *KRT19P3*, *ARHGAP27P1*, and *SFTA1P*, had decreased expression levels [56,57,58].

In hepatocellular carcinoma (HCC), higher *PDIA3P1* level is associated with poorer recurrence-free survival [59]. A group of pseudogenes, *AKR1B10P1*, *DUXAP8*, *MSTO2P*, *PDPK2P*, *SUMO1P3*, *RACGAP1P*, *ANXA2P2*, *AURKAPS1*, *PTTG3P*, and *POU5F1B*, is upregulated in HCC patient tissues and cell lines [60,61,62,63,64,65,66,67,68,69]. Higher expression of *DUXAP8* is associated with shorter OS and RFS time. Additionally, overexpression of *DUXAP8* influences the proliferation, metastasis, and EMT process [70,71]. *WFDC21P* is lower expressed in carcinoma tissues than in paired paracarcinoma tissues, and its expression levels are decreased as HCC progresses [72]. *AOC4P* (*UPAT*) is downregulated in 39.78% of individuals with HBV-related HCC [73]. *GOLGA2P10* is upregulated in HCC tissues and cells treated with ER stress inducers (tunicamycin and thapsigargin) [74]. *AKR1B10P* was found to be overexpressed in patient metastatic tissues and cell lines [60,61]. *PDIA3P1* is upregulated in multiple cancer types and following treatment with DNA-damaging chemotherapeutic agents such as doxorubicin (Dox) [59]. *RP11-424C20.2* and its parental gene *UHRF1* have elevated expression levels in patients’ liver hepatocellular carcinoma (LIHC) and thymoma (THYM) [75]. *UBE2CP3* is upregulated in patient samples and tissues with increased EV density [76].

*PDIA3P* is highly expressed in multiple myeloma (MM) and is associated with the survival rate of patients. *PDIA3P* regulates MM growth and drug resistance through Glucose 6-phosphate dehydrogenase (G6PD) along with the pentose phosphate pathway (PPP) [77].

New signatures of four pseudogenes, *RP11-326A19.5*, *RP4-706A16.3*, *RPL7AP28*, and *RPL11-551L14.1*, for osteosarcoma were found, which is a promising independent survival predictor and serves as an important biomarker for clinical treatment of osteosarcoma to improve patient management [78]. *MSTO2P* is upregulated in osteosarcoma patient samples. We found that individuals with low *MSTO2P* levels lived longer than those with increased expression. Moreover, individuals with higher stages of osteosarcoma (stage III þ IV) showed elevated expression levels of *MSTO2P* [79].

In ovarian cancer, decreased expression of *SLC6A10P* was associated with longer time to recurrence (TTR) [80]. *SDHAP1* was found to be overexpressed in patient tissues and cell lines [81]. Both *DUXAP8* and *DUXAP10* are upregulated in pancreatic carcinoma samples [82,83]. *SUMO1P3* expression was increased in pancreatic tissues compared with the corresponding adjacent normal tissues. Additionally, the data indicated that the elevated expression of *SUMO1P3* is significantly associated with tumor progression and the poor survival of individuals with pancreatic cancer. *SUMO1P3* knockdown may suppress the proliferation, migration, and invasion of pancreatic cancer cells. Furthermore, downregulation of *SUMO1P3* suppressed the epithelial–mesenchymal transition (EMT) process and not only increased the expression of epithelial cadherin but also decreased the expression of neuronal cadherin, vimentin, and β-catenin [84]. The unique feature of the *KLK4-KLKP1* fusion gene is the conversion of the non-coding *KLKP1* pseudogene into the gene encoding the protein and its unique expression in about 30% of high-grade Gleason prostate cancer [85]. All pseudogenes with diagnostic potential are summarized in Table 1.

### 3.2. Cancers Located in the Chest Area

In the cancers located in the chest area, 47 pseudogenes based on analysis of plasma-derived exosomes and tissue samples are described. Higher expression of *STXBP5*, *GALP*, and *LOC387646* indicated an unfavorable prognosis for breast cancer (BC) patients. We also found that increased *CTSLP8* and *RPS10P20* along with decreased *HLA-K* pseudogene expression indicates a poor prognosis. Pseudogene–gene interaction between *GPS2-GPS2P1* is prognostic even though neither the gene nor the pseudogene alone is prognostic of survival. *miR-3923* was predicted to target *GPS2* using miRanda, PicTar, and TargetScan, implying modules of gene–pseudogene–miRNAs that are potentially functionally related to patient survival [86]. Pseudogene *HLA-DPB2* and its parental gene *HLA-DPB1* are overexpressed and correlated with better BC patient prognosis. The *HLA-DPB2/HLA-DPB1* axis is strongly connected with immune-related biological functions. It is associated with high immune infiltration abundance of CD8+ T cells, CD4+ T cells, Tfh, Th1, and NK cells, along with elevated expression of majority biomarkers of monocytes, NK cell, T cell, CD8+ T cell, and Th1 in BC and its subtypes. It clearly indicates that *HLA-DPB2* influences the abundance of tumor-infiltrating lymphocytes in the tumor microenvironment. Additionally, *HLA-DPB2* and *HLA-DPB1* expression is positively correlated with the expression of *PD-1*, *PDL-1*, and *CTLA-4* [87].

A group of pseudogenes, *RP11-480I12.5-004*, *PCNAP1*, *PTTG3P*, *CRYβB2P1*, *CYP4Z2P*, and *PDIA3P*, was found to be upregulated in BC patients’ tissue and cell lines. Knockdown of *RP11-480I12.5* reduces cell proliferation and colony formation, induces cell apoptosis, and inhibits tumor growth in vivo. Only overexpression of *RP11-480I12.5-004* enhances cell growth both in vitro and in vivo [88]. Knockdown of *PCNAP1* suppresses the migration and invasion of cells. It also functions as a competing endogenous ceRNA for *miR-340-5p* and influences its target *SOX4*, leading to migration and invasion regulation [89]. *PTTG3P* in patients with lung adenocarcinoma (LUAD) is connected with shortening the metaphase to anaphase transition in mitosis, increasing cell viability after cisplatin or paclitaxel treatment, and facilitating tumor growth. In addition, it is associated with a poor survival rate of individuals who received chemotherapy. Knockdown of *PTTG3P* reduces cell mitosis, proliferation, and sensitivity to drugs such as paclitaxel or cisplatin [90]. *PTTG3P* is associated with BC, and it is negatively correlated with estrogen receptor (ER) and progesterone receptor (PR) status and positively related to basal-like status, triple-negative BC status, Nottingham prognostic index (NPI), and Scarff–Bloom–Richardson grade. It was indicated that its higher expression is associated with an unfavorable prognosis [91]. *CRYβB2P1* and *CRYβB2* in BC patients enhance tumorigenesis by promoting cell proliferation. Overexpression of *CRYβB2* increases invasive cellular behaviors, tumor growth, IL6 production, immune cell chemoattraction, and the expression of metastasis-associated genes [92]. Upregulation of *CYP4Z2P*-3′UTR or *CYP4Z1*-3′UTR activates signaling pathways regulating the pluripotency of stem cells, epithelial cancer stem cells, and cell cycle-related genes, and increases the CD44+/CD24− population [93,94]. Knockdown of *PDIA3P* suppresses cell viability, promotes apoptosis, and inhibits migration and invasion. *PDIA3P* negatively regulates *miR-183* and influences its target *ITGB1*, thus inducing the activation of *FAK/PI3K/AKT/β-catenin* signals and affecting tumor growth and metastasis [95].

*PTENP1* is downregulated in patient samples and cell lines, especially in advanced and more aggressive forms of BC. It regulates cell proliferation, invasion, tumorigenesis, and chemoresistance to Adriamycin (ADR). *CKS1BP7* is amplified in 28.8% of all BC patients, while *IGF1R* is amplified in 24.2% [96]. *PTENP1* activates the phosphatidylinositol-3 kinase (*PI3K*)*/AKT* pathway, and PI3K inhibitor LY294002 or siAKT prevents cancer progression [97]. *FTH1P3* is upregulated in paclitaxel-resistant BC tissue and cell lines. Knockdown of *FTH1P3* decreases the 50% inhibitory concentration value of paclitaxel, induces cell cycle arrest at the G2/M phase, and suppresses tumor growth of paclitaxel-resistant BC cells as well as ABCB1 protein expression in vivo [98].

It was found that *UGT1A1* and *BAIAP2L1* are differentially expressed between LUAD and benign lung disease [99]. *PTTG3P* and *SLC6A10P* are upregulated in LUAD patient samples. *PTTG3P* interacts with the transcription factor *FOXM1* to regulate the transcriptional activation of *BUB1B*. Moreover, it is connected with shortening the metaphase to anaphase transition in mitosis, increasing cell viability after cisplatin or paclitaxel treatment, facilitating the tumor growth, and a poor survival rate for those who received chemotherapy [91]. *SLC6A10P* is an independent prognostic factor for LUAD individuals. Its higher expression is associated with lymph node metastasis, more advanced tumor stage, and shorter overall survival in non-small cell lung cancer (NSCLC) and LUAD [100].

*LINC00908, WWC2-AS2*, and *CYP2B7P* are independent prognostic risk factors for OS, and *WWC2-AS2* with *SIGLEC17P* are independent prognostic risk factors for RFS [101]. *SUMO1P3* is upregulated in lung squamous cell carcinoma (LUSC) and LUAD patient samples. It is co-expressed with *SUMO1*, where higher *SUMO1* or *SUMO1P3* expression is associated with reduced RFS in the case of individuals with LUAD; however, only *SUMO1P3* is the independent prognostic factor. It is also correlated with late clinical stage, lymph node metastasis, distant metastasis, and a poorly differentiated degree [102,103].

A group of pseudogenes, *DUXAP8, WTAPP1, FTH1P3*, and *PDIA3P1*, was found to be upregulated in NSCLC tissue samples. *DUXAP8* expression is positively related to the cancer grade, and it influences *miR-409-3p* expression in a sponging-dependent manner and promotes HK2 as well as LDHA expression. Downregulation of *DUXAP8* inhibits tumor growth in vivo [104,105]. *WTAPP1* is negatively correlated with *HAND2-AS1*. In contrast to *HAND2-AS1,* overexpression of *WTAPP1* promotes invasion and migration [106]. Higher expression of *FTHP3* is closely correlated with worse patient prognosis due to promoting proliferation and invasion. Additionally, knockdown of *FTH1P3* represses the tumor growth in vivo [107,108]. Increased expression of *PDIA3P1* is connected with an advanced TNM, lymph node metastasis, and shorter DFS time. Knockdown of *PDIA3P* suppresses the proliferation and invasion as well as reduces tumor growth in vivo [109].

Higher expressions of *PMPCAP1* and *SOWAHC* are associated with unfavorable LUSC patient prognosis. It should be noted that *PMPCAP1*, as well as SOWAHC and ZNF454, are involved in gene expression and transcription pathways [110]. Pseudogenes described as changes in the cancers located in the chest area are listed and described in Table 1.

### 3.3. Cancers Located in the Head and Neck Area

In the case of cancers located in the head and neck area, only 37 pseudogenes have been described to date. Expression levels of Annexin 2 pseudogenes, *ANXA2-P1*, *ANXA2-P2*, *ANXA2-P3*, and *ANXA2*, were significantly increased in diffuse glioma. Meanwhile, among four glioma subtypes, it was found that *ANXA2P1*, *ANXA2P2*, and *ANXA2* are preferentially expressed in the mesenchymal subtype and less expressed in the proneural subtype [111]. *ANXA2P2* is upregulated in patient tissues and cells. It was indicated that *miR-9* has a negative correlation with the *ANXA2P2* mRNA target, and overexpression of this miRNA suppresses the cell proliferation and aerobic glycolysis of glioma cells by binding to *LDHA* 3′UTR. Knockdown of *ANXA2P2* reduces cell proliferation and aerobic glycolysis and downregulates protein levels of glycolysis markers such as *GLUT1*, *HK2*, *PFK*, and *LDHA* [112].

*LGMNP1* was found to be upregulated in glioblastoma tissues. Its high expression enhances proliferation and invasion, which leads to a more aggressive phenotype in cells overexpressing *LGMNP1*. This pseudogene functionally targets *miR-495-3p*, in a RISC-dependent manner, which targets *LGMN* (legumain, encodes a cysteine protease that has a strict specificity for hydrolysis of asparaginyl bonds) [113]. *DUXAP8* was found to be positively related to the tumor stage in neuroblastoma and is negatively associated with patient survival rate. Its knockdown reduces proliferation, colony formation, cycle, and motility [114]. In glioma and glioblastoma, *MT1JP* is downregulated in patient tissues and cell lines. Its lower expression is associated with cancer progression and poor survival. Overexpression of *MT1JP,* on the other hand, reduces proliferation and invasion [115]. *PDIA3P1* is overexpressed and its expression is connected with tumor degree and transcriptome subtype. Its increased level is correlated with unfavorable patient outcomes, as well as enhanced migration and invasion. *PDIA3P1* functions as a ceRNA by sponging *miR-124-3p* to modulate *RELA* expression and activate the downstream NF-κB pathway. HIF-1 is confirmed to directly bind to the *PDIA3P1* promoter region and activate its transcription [116].

*RPSAP52* is upregulated in patient samples, and its elevated expression is connected with shorter survival. The expression level of *RPSAP52* is positively correlated with TGF-β1, leading to its upregulation, while silencing of *RPSAP52* leads to a decrease in CD133+ cells, which seem to describe the phenotype of cancer-initiating cells [117].

Five pseudogenes, *PKMP3, AC027612.4, HILS1, RP5-1132H15.3*, and *HSPB1P1*, are identified as prognostic gene signatures. Upregulation of genes connected with phagosomes, *JAK/STAT*, *PI3K/AKT*, and *TNF* signaling pathways is observed in a high-risk group of patients divided based on five pseudogene signatures. These five pseudogenes are connected with biological processes: *PKMP3* with trans-synaptic signaling, histone modification, and *Wnt* and *MAPK* signaling pathways; *AC027612.4* with cell cycle, nuclear division, *PI3K/AKT* and *TP53* signaling pathways; *HILS1* with protein phosphorylation activity and transcriptional misregulation; *RP5-1132H15.3* with microtubule-based movement and ferroptosis; and the last one, *HSPB1P1*, with *JAK/STAT* cascade, neutrophil-mediated immunity, *TNF* signaling pathways, and apoptosis [118].

Another five pseudogenes, *ANXA2P2, EEF1A1P9, FER1L4, HILS1*, and *RAET1K*, are connected with glioma. They can be used to establish the patient risk signature. The risk signature genes are involved in regulating proliferation, migration, adhesion, ECM receptor interaction, angiogenesis, response to hypoxia (*HIF-1* signaling pathway), *PI3K/AKT* signaling pathway, and apoptosis. Additionally, increased expression of *ANXA2P2, FER1L4, HILS1*, and *RAET1K*, as well as lower levels of *EEF1A1P9* are connected with unfavorable prognosis [119].

*HERC2P2* is positively correlated with survival and negatively associated with the clinical grade of glioma. Overexpression of *HERC2P2* reduces migration and colony formation abilities and reduces tumor growth in vivo [120]. *FTH1P3* is upregulated in patient samples and cell lines. Overexpression of *FTH1P3* promotes glioma cell proliferation and inhibits apoptosis. Additionally, *FTH1P3* inhibits *miR-224-5p* expression, which in turn negatively regulates *TPD52* expression. It has been proven that the *FTH1P3/miR-224-5p/TPD52* axis is responsible for glioma progression [121]. It was indicated that *PTENP1* is downregulated in glioma patient samples. However, overexpression of *PTENP1* suppresses cell proliferation, decreases the numbers of S-phase cells, invasion, migration abilities, induces the expression of p21 protein, and suppresses the *p38* signaling pathway [122].

*AGPG* is highly expressed in many cancers. Its elevated expression levels are correlated with poor prognosis. *AGPG* is a transcriptional target of *TP53*, and loss or mutation of *TP53* induces upregulation of *AGPG*. It was shown that AGPG protects PFKFB3 from proteasomal degradation and leads to the accumulation of PFKFB3, which activates glycolytic flux and promotes cell cycle progression. In esophageal squamous cell carcinomas (ESCC), knockdown of *AGPG* results in tumor growth in patient-derived xenograft models [123].

Another group of five pseudogenes in head and neck squamous cell carcinoma (HNSCC), *LILRP1, RP6-191P20.5, RPL29P19, TAS2R2P*, and *ZBTB45P1*, can be used as prognostic or predictive markers. Signatures of these five pseudogenes can distinguish the low-risk and high-risk individuals, predicting prognosis with high sensitivity and specificity. This group is associated with the immune system and cancer-related biological processes. *LILRP1* and *RP6-191P20.5* are involved in immune regulation, *PRL29P19* in metabolism regulation, and *TAS2R2P* and *ZBTB45P1* have multiple functions, and also in various pathways enriched in the high-risk group such as EMT process, angiogenesis, metastasis, proliferation, extracellular matrix receptor, focal adhesion, and *PI3K/AKT* pathways [124].

Another marker in HNSCC is *PTTG3P*. It is upregulated in patient samples, its expression depends on the type of mutation in the *TP53* gene, and it correlates with genes from the *TP53* pathway. Patients with low expressions of *PTTG3P* have longer DFS time. Furthermore, expression levels of *PTTG3P* depend on T-stage, grade, and HPV p16 status. Interestingly, the *PTTG3P* high-expressing group of patients have the most dysregulated genes connected with DNA repair, oxidative phosphorylation, and peroxisome pathways [125].

A double homeobox A pseudogene 10 (*DUXAP10*) can be used as a marker in both oral squamous cell carcinoma (OSCC) and ESCC. A total of 4462 DEGs and 76 differentially expressed lncRNAs were screened between the three groups, and 200 DEGs and only *DUXAP10* were screened among the three groups. A total of 1662 interactions of 46 lncRNAs and their coexpressed target genes were predicted, and 38 pairs of lncRNA-lncRNA coregulated 843 target genes. The coregulated target genes are significantly enriched in the antigen adaptive immune response, activation of phagocytosis receptor signaling, or mast granule *NF-κB* inflammation. Overall, lncRNAs were differentially expressed in OSCC and dysplasia. The target genes might play an essential role in the carcinogenesis and development of OSCC. These results improve our understanding of the lncRNA-based pathogenesis and identify potential targets for early diagnosis of malignant transformation from dysplasia to OSCC. *DUXAP10* was certified to be upregulated in ESCC tissues and cells. Additionally, it was positively correlated with a short survival time. Moreover, the down-expression of this pseudogene contributed to decreased cell proliferation and metastasis. Silenced *DUXAP10* led to increased apoptosis rate and stagnation of the cell cycle. Results of mechanistic 196 experiments suggested that *DUXAP10* motivated ESCC progression through recruiting enhancer of zeste homolog 2 (*EZH2*) to the promoter of p21 [126].

*FKBP9P1* is upregulated in patient tissues, as well as cell lines, and its elevated level is correlated with advanced T-stage, N-stage, and clinical stage, and it is connected with a shorter OS and DFS time. Knockdown of *FKBP9P1* reduces proliferation, migration, and invasion by reducing the *PI3K/AKT* signaling pathway activity. *FTH1P3* is upregulated in ESCC patients’ samples. It was indicated that higher *FTH1P3* expression is connected with a worse prognosis. Overexpression of *FTH1P3* increases cell proliferation, migration, and invasion, and inhibits cell apoptosis. It is also positively correlated with poorer differentiation, increased T classification, lymph node metastasis, and advanced clinical stage [127]. *FTH1P3* is also upregulated in OSCC and ESCC patient samples and cell lines. The expression level of *FTH1P3* was significantly upregulated in OSCC tissues and cell lines. Increased expression of *FTH1P3* in OSCC tissue was associated with T classification, N classification, and TNM stage. Furthermore, Kaplan–Meier survival analysis proved that the prognosis of individuals with low *FTH1P3* expression was much better than for those with high expression. Cox regression analysis showed that *FTH1P3* expression was an independent prognosis-predicting factor for individuals with OSCC. Loss-function assay indicated that knockdown of *FTH1P3* significantly suppressed the proliferation, migration, and invasion of OSCC cells. Mechanistically, we found that knockdown of *FTH1P3* significantly reduced the activation of *PI3K/AKT/GSK3β/Wnt/β-catenin* signaling [128].

The last one, *TUSC2P*, is downregulated in patient samples and cell lines. Its elevated expression is associated with better patient survival. *TUSC2P*-3′UTR regulates the expression of *miR-17-5p*, *miR-520a-3p*, *miR-608*, and *miR-661* in a sponging-dependent manner and protects *TUSC2* mRNA from being regulated by these miRNAs [129,130]. All pseudogenes with diagnostic potential are summarized in Table 1.

**Table 1:** Pseudogenes with potential biomarker utility in cancers in chosen locations.

Name of Biomarkers	Location of Cancer	Type of Cancer	Type of Biomarker	Determination Method	Type of Sample	Description/Function	Ref.
*RP4-706A16.3*	Abdomen and bones	Osteosarcoma	predictive	Analyzed RNA-seq	tissue	signature of four (*RP11-326A19.5, RP4-706A16.3, RPL7AP28, RPL11-551L14.1*) pseudogenes for osteosarcoma, which is a promising independent survival predictor and serves as an important biomarker for clinical treatment of osteosarcoma to improve patient management	[78]
fusion gene *KLK4-KLKP1*		Prostate Cancer	diagnostic	Urine samples, fusion can also be detected in needle biopsy tissue samples using a specific antibody	urine	the unique feature of this fusion gene is the conversion of the non-coding *KLKP1* pseudogene into the gene encoding the protein and its unique expression in about 30% of high-grade Gleason prostate cancer	[85]
*GBP1P1* and *PTTG3P*	Abdomen and bones	Cervical Carcinoma	diagnostic	Microarray analysis and qRT-PCR of patient samples and cell lines	tissue	8 overexpressed transcribed pseudogenes (*GBP1P1, HLA-DRB6, HLA-H, SLC6A10P, NAPSB, KRT16P2, PTTG3P*, and *RNF126P1*) and 2 overexpressed pseudogenes (*GBP1P1* and *PTTG3P*) observed in microarray analysis and validated by qRT-PCR	[46]
*MSTO2P*	Abdomen and bones	Osteosarcoma	prognostic	qRT-PCR of patient samples	tissue	it is upregulated in patient samples patients with low *MSTO2P* levels lived longer than those with high *MSTO2P* levels patients with higher stages of osteosarcoma (stage III þ IV) showed higher expression levels of *MSTO2P* knockdown of MSTO2P reduces cell growth, invasion, and EMT of osteosarcoma cells under hypoxia conditions *PD-L1* acts as a key effector for *MSTO2P*-regulated osteosarcoma progression under hypoxia conditions *MSTO2P* positively influences the tumor growth in immunodeficient mice and in the human clinical tissues	[79]
*PA2G4P4*	Abdomen and bones	Bladder Cancer	diagnostic	qRT-PCR and ISH of patient samples	tissue	it is overexpressed in patient tissues and in cell lines *PA2G4P4* distribution strictly overlaps *PA2G4/EBP1* protein localization knockdown of *PA2G4P4* affects proliferation and migration of cells	[45]
*FTH1P3*	Abdomen and bones	Cervical Cancer	diagnostic	qRT-PCR of patient samples and cell lines	tissue	it is upregulated in patient samples and cell lines knockdown of *FTH1P3* reduces cell proliferation, invasion, and migration, and promotes apoptosis *miR-145* is a direct target of *FTH1P3* and has effects on cell viability and mobility	[47]
*BMI1P1*	Abdomen and bones	Acute Myeloid Leukemia	diagnostic and prognostic	qRT-PCR of patient samples	tissue	*BMI1P1A* and *OCT4* and *POU5F1B* make up a three-gene signature that divides patients into high-risk and low-risk groups the three-gene signature is a more valuable signature for distinguishing between patients and controls than any of the three genes the three-gene signature was a prognostic factor: high-risk patient group has shorter leukemia-free survival (LFS) OS than the low-risk group	[44]
*EMBP1*	Abdomen and bones	Renal Cell Carcinoma	diagnostic	qRT-PCR of patient samples and cell lines	tissue	it is upregulated in patient samples and cell lines correlation of *EMBP1* with clinicopathological characteristics of patients knockdown of *EMBP1* reduces proliferation, migration, and invasion, and promotes apoptosis and cell cycle arrest *EMBP1* directly binds to and negatively regulates *miR-9-5p;* *EMBP1-miR-9-5p* axis influences EMT (changes in expression of *E-cadherin, claudin, vimentin, KLF4, Nanog*) and the cell cycle (changes in expression of *CCNE2*) and its downstream mediator *E2F1* *miR-9-5p* overexpression or *EMBP1* upregulation reduces xenograft tumor growth in vivo, effects that are abrogated by *CCNE2* overexpression	[131]
*DUXAP8* and *DUXAP9*	Abdomen and bones	Renal Cell Carcinoma	diagnostic and prognostic	RNA-seq (TCGA data)	tissue	higher expression of *DUXAP8* and *DUXAP9* is connected with poorer patient prognosis 33 and 5 miRNAs are predicted to potentially bind to *DUXAP8* and *DUXAP9*, respectively *miR-29c-3p* has the most potential as a binding miRNA of *DUXAP8* and *DUXAP9* *COL1A1* and *COL1A2* are targets of *DUXAP8* and *DUXAP9*, and are regulated by *miR-29c-3p* *DUXAP8* and *DUXAP9* enhances but *miR-29c-3p* weakens the carcinoma growth	[132]
*POU5F1B*	Abdomen and bones	Cervical Cancer	diagnostic	qRT-PCR of patient samples and cell lines	tissue	it is upregulated in patient tissues and cell lines knockdown of *POU5F1B* inhibits cell proliferation, apoptosis, migration, and invasion, as well as tumor growth in vivo it modulates the expression of the OCT4 protein and influence on cell phenotype	[48]
*DUXAP8*	Abdomen and bones	Pancreatic Carcinoma	diagnostic and prognostic	GEO databases (GSE16515, GSE15932, GSE15471) and qRT-PCR of patient samples and cell lines	tissue	it is upregulated in patient samples higher expression is associated with a larger tumor size, advanced pathological stage higher expression is associated with shorter OS time knockdown of *DUXAP8* inhibits cell proliferation and promotes apoptosis *DUXAP8* regulates cell proliferation partly through downregulation *CDKN1A* and *KLF2*	[82]
*PTENP1*	Abdomen and bones	Endometrial Hyperplasia and Carcinomas	diagnostic	Methyl-sensitive PCR of genomic DNA	tissue/blood	it is methylated in all analyzed tissues, except for the peripheral blood; no differences between the EC and EH groups methylation level was higher in patients than controls (71–77% vs. 58%) *PTENP1* pseudogene methylation is age-related and is not directly related to the endometrium pathology under study methylation may protect against the development of EC and/or serve as a marker of a precancerous condition of endometrial cells	[54]
*DUXAP10*	Abdomen and bones	Pancreatic Cancer	diagnostic	GEO databases (GSE15471, GSE15932, GSE16515) and qRT-PCR of patient samples and cell lines	tissue	it is upregulated in patient samples expression of *DUXAP10* is higher in patients with an advanced TNM stage and positive lymph node metastasis higher expression of *DUXAP10* positively influences cell cycle progression, cell growth, migration, and invasion, and reduces apoptosis *DUXAP10* regulates cell proliferation through interacting with RNA-binding proteins EZH2 and LSD1	[83]
*CEACAM22P, MSL3P1, TREML3P*	Abdomen and bones	Renal Cell Carcinoma	diagnostic and prognostic	RNA-seq (TCGA data) and qRT-PCR of patient samples	tissue/serum	2553 lncRNAs and 8901 pseudogenes are changed and occurred in up to 23% of all cases 27 lncRNAs and 45 pseudogenes are connected with patient prognosis pseudogenes *CEACAM22P, MSL3P1*, and *TREML3P* (and lncRNAs *LINC00520, PIK3CD-AS1*, and *LINC01559*) can be used as non-invasive serum based biomarkers only upregulation of *PIK3CD-AS1* is associated with higher tumor stage and metastasis	[133]
*DUXAP8*	Abdomen and bones	Renal Cell Carcinoma	diagnostic and prognostic	qRT-PCR of patient samples and cell lines	tissue	it is upregulated in patient tissues and cell lines knockdown of *DUXAP8* reduces cell proliferation and invasion *DUSP8* downregulation of *miR-126* expression, which targets *CED-9* (apoptosis regulator) and influences cell proliferation	[134]
*PDIA3P*	Abdomen and bones	Multiple Myeloma	diagnostic, prognostic and predictive	qRT-PCR of patient samples and cell lines	tissue	highly expressed in MM and is associated with the survival rate of MM patients *PDIA3P* regulates MM growth and drug resistance through glucose 6-phosphate dehydrogenase (G6PD) and the pentose phosphate pathway (PPP) *PDIA3P* interacts with c-Myc to enhance its transactivation activity and binding to *G6PD* promoter, stimulating *G6PD* expression and PPP flux *PDIA3P* is overexpressed in U266 cells in the presence of bortezomib and overexpression of *PDIA3P* restored the inhibitory effect of bortezomib on cell proliferation	[77]
*SUMO1P3*	Abdomen and bones	Pancreatic Cancer	diagnostic and prognostic	qRT-PCR of patient samples and cell lines	tissue	*SUMO1P3* expression was elevated in pancreatic tissues compared with the corresponding adjacent normal tissues the data indicated that the increased expression of *SUMO1P3* is significantly associated with tumor progression and the poor survival of patients with pancreatic cancer *SUMO1P3* knockdown may suppress the proliferation, migration, and invasion of pancreatic cancer cells downregulation of *SUMO1P3* suppressed the EMT and increased the expression of epithelial cadherin, and decreased the expression of neuronal cadherin, vimentin, and β-catenin	[84]
*SLC6A10P*	Abdomen and bones	Ovarian Cancer	predictive	Analyze original RNA-seq; microarray analysis of primary tumors identified genes that may be useful in risk stratification/overall survival but have limited value in predicting >70% tumor recurrence rates	tissue	recurrence of the tumor, after an initial response to adjuvant chemotherapy, is a serious problem in women with high-grade serous ovarian cancer (HGSOC) identified genes that may be useful in risk stratification	[80]
*HMGA1P6*	Abdomen and bones	Ovarian Cancer	prognostic	Microarray analysis of patient samples and TCGA analysis	tissue	identification of 577 dysregulated pseudogenes; 538 of them are upregulated *HMGA1P6* is overexpressed and its expression is inversely correlated with patient survival *HMGA1P6* promoted cell malignancy by acting as a ceRNA by enhancing HMGA1 and HMGA2 expression *HMGA1P6* is transcriptionally activated by oncogene MYC	[135]
*LDHAP5*	Abdomen and bones	Ovarian Serous Cystadenocarcinoma	diagnostic, prognostic and predictive	RNA-seq (TCGA/dreamBase)	tissue	identification of 63 upregulated pseudogenes *LDHAP5* is connected with shorter OS time connected with pathways involved with miRNA in cancer, pathways in cancer, and *PI3K/AKT* pathway *EGFR* is the potential targeted mRNA by *LDHAP5*	[136]
*SDHAP1*	Abdomen and bones	Ovarian Cancer	diagnostic, prognostic and predictive	qRT-PCR of patient samples and cell lines	tissue	it is overexpressed in patient tissues and cell lines knockdown of *SDHAP1* induces re-acquirement of chemo-sensitivity to PTX in ovarian cancer cells in vitro *SDHAP1* upregulates the expression of *EIF4G2* by *miR-4465* in a sponging-dependent manner, and by this way, influences chemosensitivity	[81]
*DUXAP8, RP11-54H7.4,* and *RP11-138J23.1*	Abdomen and bones	Colon Cancer	diagnostic and prognostic	RNA-seq (TCGA data)	tissue	*DUXAP8, RP11-54H7.4* and *RP11-138J23.1* show higher expression in advanced tumor stages higher expression of *DUXAP8* (as well as *ELFN1-AS1*) is connected with poor prognosis	[49]
*REG1CP*	Abdomen and bones	Colorectal Cancer	diagnostic and prognostic	qRT-PCR, ddPCR and ISH of patient samples and cell lines; databases	tissue	it is upregulated in patient samples and cell lines upregulation of *REG1CP* is an early event during colorectal tumorigenesis *REP1CP* levels are higher in colon adenomas and dysplastic colon mucosa and in colon cancers compared to normal mucosa higher level is associated with poorer PFS *REG1CP* activates *REG3A* by forming an RNA–DNA triplex with the *REG3A* gene	[51]
*KCNQ1OT1*	Abdomen and bones	Colorectal Cancer	diagnostic and prognostic	RNA-seq (TCGA data), GEO databases (GSE14333, GSE39582, GSE103479) and qRT-PCR of patient samples	tissue	higher *KCNQ1OT1* (as well as *PNN*) is associated with shorter DFS in stage III patients treated using 5-FU adjuvant therapy no difference was observed in the case of untreated patients	[50]
*TPTE2P1*	Abdomen and bones	Colorectal Cancer	diagnostic and prognostic	qRT-PCR of patient samples and cell lines	tissue	it is upregulated in patient samples higher expression is associated with a worse survival rate knockdown of *TPTE2P1* leads to cell cycle arrest (S phase), inhibits cell viability, induces cell apoptosis by the *BCL2/caspase 3* signaling activation reduction of *TPTE2P1* has suppressive effects on tumors in vivo	[52]
*DUXAP8*	Abdomen and bones	Colorectal Cancer	diagnostic and prognostic	RNA-seq (TCGA data) and qRT-PCR of patient samples and cell lines	tissue	it is upregulated in patient samples higher expression connected with advanced clinical progression and poor survival STAT3 is responsible for the upregulation of *DUXAP8* knockdown of *DUXAP8* inhibits cell proliferation, migration, and invasion, and promotes apoptosis *DUXAP8* regulates the expression of *miR-577* as competing endogenous RNA and modulates expression of *RAB14*	[53]
*PMS2L2*	Abdomen and bones	Gastric Adenocarcinoma	diagnostic and prognostic	qRT-PCR of patient samples and cell lines	tissue	it is downregulated in patient tissues, and it does not depend on clinical stage lower expression is associated with a lower OS time *miR-25* is inversely correlated with *PMS2L2* overexpression of *PMS2L2* reduces the expression of *miR-25* overexpression of *PMS2L2* inhibits migration and invasion	[55]
*KRT19P3*	Abdomen and bones	Gastric Cancer	diagnostic and prognostic	Microarray and qRT-PCR of patient samples and cell lines	tissue	it is downregulated patient tissues and cell lines lower expression is correlated with larger tumor size, advanced TNM stage, Lauren’s classification, positive lymph node metastasis; lower expression is connected with poor prognosis upregulation of *KRT19P3* inhibits cell proliferation, migration, and invasion in vitro, as well as tumorigenesis and metastasis in vivo *KRT19P3* directly binds to *COPS7A*, regulates it expression, and suppress tumor growth and metastasis through *COPS7A*-mediated *NF-κB* pathway	[57]
*ARHGAP27P1*	Abdomen and bones	Gastric Cancer	diagnostic and prognostic	qRT-PCR of patient samples and cell lines	tissue and plasma	it is downregulated in patient tissues, plasma, and cell lines lower expression is associated with advanced TNM stage, increased invasion depth, and lymphatic metastasis lower expression is connected with a poor prognosis overexpression of *ARHGAP27P1* inhibits proliferation, invasion, and migration *ARHGAP27P1* is associated with *JMJD3* and that this association is required for the demethylation of 3K27me3, thereby epigenetically activating expression of *p15, p16* and *p57* knockdown of *JMJD3, p15* or *p16* reverses the inhibitory effects of *ARHGAP27P1* in cell proliferation and cell cycle progression	[58]
*SFTA1P*	Abdomen and bones	Gastric Cancer	diagnostic and prognostic	qRT-PCR of patient samples and cell lines	tissue	it is downregulated in patient samples decreased expression is correlated with advanced TNM stage, larger tumor size, lymphatic metastasis lower expression is associated with poorer prognosis overexpression of *SFTA1P* inhibits cell proliferation, migration, and invasion downregulation of *SFTA1P* is associated with decreased *TP53* expression	[56]
*DUXAP10*	Abdomen and bones	Gastric Cancer	diagnostic and prognostic	GEO database (GSE54129, GSE70880, GSE79973, and GSE99416) and qRT-PCR of cell lines	tissue	it is upregulated in patient samples and cell lines higher expression is associated with poor patient prognosis knockdown of *DUXAP10* inhibits cells proliferation, migration, and invasion *DUXAP10* interacts with *PRC2* and *LSD1* and represses *LATS1* expression at transcriptional level, and binds with *HuR* to maintain the stability of *β-catenin* mRNA and increase its protein levels	[137]
*PDIA3P1*	Abdomen and bones	Hepatocellular Carcinoma	predictive	real-time quantitative PCR of patient samples	tissue	higher *PDIA3P1* level is associated with poorer recurrence-free survival protection of cancer cells from Dox-induced apoptosis	[59]
*HSPB1P1*	Abdomen and bones	Hepatocellular Carcinoma	prognostic	RNA-seq from GSE124535 dataset	tissue	identified of 16 up- and 17 downregulated pseudogenes *HSPB1P1* is abnormally expressed in 20 types of cancers can be used as an indicator for poorer overall survival of patients with HCC *HSPB1P1* is strongly correlated with signaling pathways related to cancer progression and direct regulates the *EZH2* expression	[138]
*AKR1B10P1*	Abdomen and bones	Hepatocellular Carcinoma	diagnostic	RNA-seq (TCGA data) and microarray analysis (GEO), qRT-PCR of patient samples and cell lines	tissue	it is upregulated in patient samples and cell lines positively correlated with *AKR1B10* *AKR1B10P1* is connected with larger tumor size, more advanced TNM stages, higher serum Alpha-fetoprotein (AFP) quantity, tumor microsatellite formation, and liver cirrhosis knockdown of *AKR1B10P1* reduces cell proliferation, induces cell cycle arrest and cell apoptosis, and impairs the ability of cell mobility *AKR1B10P1* influences EMT by directly interacting with *miR-138* which regulates *SOX4*, a pivotal promotor of EMT	[60]
*DUXAP8*	Abdomen and bones	Hepatocellular Carcinoma	diagnostic and prognostic	qRT-PCR of patient samples and cell lines	tissue	it is upregulated in patient tissues it is upregulated in Stage II/III compared to Stage I samples higher expression of *DUXAP8* is associated with shorter OS and RFS time knockdown of *DUXAP8* reduces proliferation and induces apoptosis *DUXAP8* regulates multiple cell cycle regulators such as promotes *BUB1* expression by sponging-mediated suppression of *miR-490-5p*	[70]
Panel of pseudogenes *(ABCC6P2, ANXA2P2, AQP7P1, AZGP1P1, C3P1, CA5BP1, DSTNP2, HLA-J, HSPA7, LPAL2, NAPSB, NUDT16P1, PLGLA, RP9P)*	Abdomen and bones	Hepatocellular Carcinoma	diagnostic and prognostic	RNA-seq (TCGA data)	tissue	establishment of 19 pseudogene pair signatures, which included 21 pseudogenes (*ABCC6P2, ANXA2P2, AQP7P1, AZGP1P1, C3P1, CA5BP1, DSTNP2, HLA-J, HSPA7, LPAL2, NAPSB, NUDT16P1, PLGLA, RP9P*) patients in high-risk group have an increased risk of worse prognosis pseudogenes are primarily involved in cytokine receptor activity, T cell receptor signaling, chemokine signaling, *NF-κB* signaling, *PD-L1* expression, and the *PD-1* checkpoint pathway in cancer	[139]
*AOC4P (UPAT)*	Abdomen and bones	Hepatocellular Carcinoma	diagnostic, prognostic and predictive	qRT-PCR of patient samples and cell lines	tissue	it is downregulated in 39.78% of patients with HBV-related HCC low level of *UPAT* was associated with multiple worse clinicopathological parameters and shorter RFS time overexpression of *UPAT* suppresses cellular migration, invasion, EMT processes, and CSC properties *UPAT* is negatively correlated with ZEB1 protein *UPAT* promotes ZEB1 degradation via a ubiquitin-proteasome pathway and in turn ZEB1 transcriptionally suppresses *UPAT* by binding to multiple E-box (CACCTG) elements in the promoter region	[73]
*WFDC21P*	Abdomen and bones	Hepatocellular Carcinoma	diagnostic, prognostic and predictive	qRT-PCR of patient samples and cell lines	tissue	it is lower expressed in carcinoma tissues than in paired paracarcinoma tissues and its expression levels are decreased as HCC progress high expression is connected with longer OS time *WFDC21P* reduces glycolysis by simultaneously interacting with PFKP and PKM2 (two key enzymes in glycolysis) by influencing abrogate the tetramer formation of PFKP to impede its catalytic activity and by preventing the nuclear translocation of PKM2 to suppress its function as a transcriptional coactivator *WFDC21P* expression is positively correlated with Nur77 Nur77 binds to its response elements on the *WFDC21P* promoter to directly induce *WFDC21P* transcription, which inhibits HCC cell proliferation, tumor growth, and tumor metastasis cytosporone-B (an agonist for Nur77) stimulates *WFDC21P* expression and suppress cancer in a *WFDC21P*-dependent manner	[72]
*DUXAP8*	Abdomen and bones	Hepatocellular Carcinoma	diagnostic and prognostic	qRT-PCR of patient samples and cell lines	tissue	it is upregulated in patient samples correlated with unfavorable pathological features higher expression is associated with shorter OS time overexpression influences the proliferation, metastasis, and EMT knockdown of *DUXAP8* reduces the malignant phenotype *DUXAP8* interacts with *miR-422a* in sponging-dependent manner and enhances the expression of *PDK2*	[71]
*GOLGA2P10*	Abdomen and bones	Hepatocellular Carcinoma	diagnostic and prognostic	qRT-PCR of patient samples and cell lines	tissue	it is upregulated patient tissues and in cells treated with ER stress inducers (tunicamycin and thapsigargin) higher expression is correlated with shorter RFS time *GOLGA2P10* increased BCL-xL protein level, promoted BAD phosphorylation, and conferred tumor cells with resistance to ER stress-induced apoptosis upon ER stress, CHOP directly bound to the promoter of *GOLGA2P10* and induced its transcription via the *PERK/ATF4/CHOP* pathway, which protects tumor cells from the cytotoxic effect of persistent ER stress in tumor microenvironment by regulating *Bcl-2* family members the ER stress inducer-stimulated apoptosis is induced by silencing *GOLGA2P10* and reduced by its overexpressing	[74]
*MSTO2P*	Abdomen and bones	Hepatocellular Carcinoma	diagnostic and prognostic	RNA-seq (TCGA data), dataset GSE30219, and qRT-PCR of patient samples and cell lines	tissue	it is upregulated in patient tissues and cells lines *MSTO2P* increases cell proliferation, invasion, and metastasis knockdown of *MSTO2P* has influence on EMT process by increasing E-cadherin and decreasing N-cadherin and vimentin expressions *MSTO2P* increases the expressions of proteins in the *PI3K/AKT/mTOR* pathway, including PI3K, p-AKT, and p-mTOR	[62]
*AKR1B10P*	Abdomen and bones	Hepatocellular Carcinoma Cells	diagnostic	qRT-PCR of patient samples and cell lines	tissue	it is overexpressed in patient metastatic tissues and cell lines positively correlated with its parental genes high level is correlated with the worst clinicopathologic features (with larger tumor dimension, higher level of AFP, advanced TNM stages, tumor microsatellite formation and venous invasion) SOX4 activates the *AKR1B10P1* transcription positive feedback between *AKR1B10P1* and *miR-138* by competing endogenous RNA (ceRNA) way *AKR1B10P1/miR-138/SOX4* axis promotes cell proliferation	[61]
*PDIA3P1*	Abdomen and bones	Hepatocellular Carcinoma and Multiple Cancer Types	diagnostic, prognostic and predictive	qRT-PCR of patient samples and cell lines, data sets GSE43541, GSE58074, GSE32301, GSE42531, GSE63351 for cell line	tissue	is upregulated in multiple cancer types and following treatment with DNA-damaging chemotherapeutic agents (doxorubicin, Dox) higher level is associated with poorer RFS of human hepatocellular carcinoma *PDIA3P1* protects cancer cells from Dox-induced apoptosis and allows tumors to grow faster and to be more resistant to Dox in vivo *PDIA3P1* binds to *miR-125a/b/miR-124* in sponging-dependent manner and reduces their level and it in turn represses *TRAF6*, leading to activation of the *NF-κB* pathway administration of BAY 11-7085 (an NF-κB inhibitor) reduces *PDIA3P1*-dependent resistance to doxorubicin upregulation of PDIA3P1 is correlated with elevation of TRAF6, phosphorylated p65, and NF-κB downstream anti-apoptosis genes hMTR4 (which promotes RNA degradation) binds to *PDIA3P1* but this interaction is disrupted by Dox treatment, and in this way, the resistance is created	[59]
*PDPK2P*	Abdomen and bones	Hepatocellular Carcinoma	diagnostic and prognostic	Microarray and qRT-PCR of patient samples and cell lines	tissue	it is upregulated in patient tissues upregulation is associated with a larger tumor embolus, low differentiation, and poor survival *PDPK2P* interacted with *PDK1* and promotes progression through the *PDK1/AKT/caspase 3* signaling pathway	[63]
*SUMO1P3*	Abdomen and bones	Hepatocellular Carcinoma	diagnostic, prognostic and predictive	qRT-PCR of patient samples and cell lines	tissue	it is upregulated in patient tissues and cell lines highly expressed in patients with higher TNM stage knockdown of *SUMO1P3* suppresses cell proliferation, colony formation ability, and cell invasiveness, and promotes apoptosis and enhances radiosensitivity	[64]
*RP11-424C20.2*	Abdomen and bones	Liver Hepatocellular Carcinoma And Thymoma	diagnostic, prognostic and predictive	RNA-seq (TCGA data)	tissue	its parental gene *UHRF1* is upregulated in liver hepatocellular carcinoma (LIHC) and thymoma (THYM) higher expressions of *RP11-424C20.2* or *UHRF1* are associated with worse patient survival for LIHC and THYM patients *RP11-424C20.2* acts as a competing endogenous RNA (ceRNA) to increase *UHRF1* expression through regulation of *miR-378a-3p* in a sponging-dependent manner *UHRF1* is connected with immune-related biological (immune infiltration, and different types of tumor-infiltrating immune cells displayed different impacts on clinical outcomes) *UHRF1* expression in LIHC and THYM shows an opposite correlation with biomarkers from monocyte, dendritic cell, Th1, and T cell exhaustion *RP11-424C20.2/UHRF1* axis regulates immune escape of LIHC and THYM, partly through IFN-γ-mediated *CLTA-4* and *PD-L1* pathway	[75]
*RACGAP1P*	Abdomen and bones	Hepatocellular Carcinoma	diagnostic and prognostic	Microarray and qRT-PCR of patient samples and cell lines, datasets GSE84005, GSE76297, GSE6404, GSE54236, and GSE5975 and TCGA	tissue	it is upregulated in patient samples it is associated with larger tumor size, advanced clinical stage, abnormal AFP level, and shorter survival time *RACGAP1P* regulates the development of malignant characteristics of cells, including cell growth and migration *RACGAP1P* acts as a ceRNA and reduces *miR-15-5p* leading to the upregulation of *RACGAP1* and the activation of *RhoA/ERK* signaling	[65]
*ANXA2P2*	Abdomen and bones	Hepatocellular Carcinoma	diagnostic and prognostic	RNA-seq (TCGA data) and qRT-PCR of patient samples and cell lines	tissue	it is upregulated in patient samples higher *ANXA2P2* expression is connected with shorter OS time independent from clinical parameters, such as age, gender, histological grade, T classification, stage, albumin level, alpha-fetoprotein, and vascular invasion knockdown of *ANXA2P2* inhibits migration and invasion	[66]
*AURKAPS1*	Abdomen and bones	Hepatocellular Carcinoma	diagnostic	qRT-PCR of patient samples and cell lines	tissue	it is upregulated in patient samples higher expression is associated with tumor size and TNM stage *AURKAPS1* promotes cell movement, migration, and invasion *AURKAPS1* regulates the expression of *miR-182*, *miR-155* and *miR-14* and increases the protein expression of RAC1, promotes the activation of ERK, and enhances the formation of membrane ruffles	[67]
*UBE2CP3*	Abdomen and bones	Hepatocellular Carcinoma	diagnostic and prognostic	qRT-PCR and ISH of patient samples and cell lines	tissue	it is upregulated in patient samples and in tissues with high EV density overexpression of *UBE2CP3* promotes proliferation, migration, and tube formation via the activation of *ERK/HIF-1α/p70S6K/VEGFA* signaling and increases the level of VEGFA	[76]
*PTTG3P*	Abdomen and bones	Hepatocellular Carcinoma	diagnostic and prognostic	Microarrays of patient samples, qRT-PCR and ISH of patient samples and cell lines	tissue	it is upregulated in patient samples it is positively correlated with tumor size, TNM stage, and poor survival overexpression of *PTTG3P* promotes cell proliferation, migration, and invasion in vitro, as well as tumorigenesis and metastasis in vivo over-expression of *PTTG3P* upregulates *PTTG1* and activates *PI3K/AKT* signaling and influences cell cycle progression, cell apoptosis and EMT	[68]
*POU5F1B*	Abdomen and bones	Hepatocellular Carcinoma	diagnostic and prognostic	RNA-seq (TCGA data) and qRT-PCR of cell lines	tissue	it is upregulated in patient samples and cell lines higher expression of *POU5F1B* is associated with shorter survival knockdown of *POU5F1B* inhibits proliferation, cell cycle progression, and colony formation in soft agar *POU5F1B* is positively correlated with *AKT* and, by activation of *AKT* influences cell phenotype	[69]
*UGT1A1, BAIAP2L1, LOC100129096, PTMAP2, CDC14C, LOC643634, FTH1P2, ARPC3P3, FTH1P11, PTMAP5*	Chest area	Lung Adenocarcinoma	diagnostic	RNA-seq	plasma-derived exosomes	*UGT1A1* and *BAIAP2L1* are differentially expressed between lung adenocarcinoma benign lung disease *LOC100129096, PTMAP2, CDC14C, LOC643634, FTH1P2, ARPC3P3, FTH1P11* and *PTMAP5* are observed in plasma-derived exosomes in lung adenocarcinoma patients, more abundant/detectable than in healthy volunteers	[99]
*PTTG3P*	Chest area	Lung Adenocarcinoma	diagnostic, prognostic and predictive	Microarray gene profiling datasets: (GSE27262, GSE31210, GSE30219 and GSE19188) containing both the tumor and normal tissue samples. Six datasets (GSE31210, GSE50081, GSE37745, GSE30219, GSE3141 and GSE19188) and RNA-seq TCGA	tissue	it is upregulated in patient samples shortens the metaphase to anaphase transition in mitosis, increases cell viability after cisplatin or paclitaxel treatment, facilitates tumor growth associated with a poor survival rate of patients who received chemotherapy *PTTG3P* interacts with the transcription factor *FOXM1* to regulate the transcriptional activation of *BUB1B* knockdown of *PTTG3P* reduces cell mitosis, proliferation, and drug sensitivity (paclitaxel or cisplatin)	[90]
*WTAPP1*	Chest area	Non-Small-Cell Lung Carcinoma	diagnostic and prognostic	qRT-PCR of patient samples and cell lines	tissue	it is upregulated in patient samples low plasma level is connected with better survival rate *WTAPP1* is negatively correlated with *HAND2-AS1* overexpression of WTAPP1 results in downregulation of *HAND2-AS1*, while overexpression of *HAND2-AS1* does not influence the *WTAPP* expression overexpression of *WTAPP1* promotes, in contrast to *HAND2-AS1*, invasion and migration overexpression of *HAND2-AS1* partially reduces the effects of *WTAPP1*	[106]
*FTH1P3*	Chest area	Non-Small-Cell Lung Carcinoma	diagnostic, prognostic and predictive	RNA-seq (TCGA data) and qRT-PCR of patient samples and cell lines	tissue	it is upregulated in the gefitinib-resistant cells higher expression is closely correlated with worse patient prognosis it promotes the proliferation and invasion and knockdown of *FTH1P3*, represses the tumor growth in vivo transcription factor E2F1 accelerates the transcription of *FTH1P3* *FTH1P3* recruits LSD1 and epigenetically represses the *TIMP3*, which leads to the tumorigenesis	[108]
*AOC4P*	Chest area	Non-Small-Cell Lung Carcinoma	diagnostic	RNA-seq (TCGA data) and qRT-PCR of cell lines	tissue	it is downregulated in patient samples and cell lines overexpression of *AOC4P* reduces viability, invasion, the expression of *MMP-2* and *MMP-9*, apoptosis and caspase-3/7 activity *AOC4P* overexpression suppresses tumor growth in vivo the activation of the *Wnt/β-catenin* pathway by BML-284 reduces the effects of *AOC4P* overexpression	[140]
*TPTEP1*	Chest area	Non-Small-Cell Lung Carcinoma	diagnostic and prognostic	RNA-seq (TCGA data), dataset GSE30219, and qRT-PCR of patient samples and cell lines	tissue	it is downregulated in patient samples expression is lower in high-grade (stage III–IV) tumors compared with low-grade tumors (stage I–II) higher expression is associated with a longer OS time overexpression of *TPTEP1* reduces cell proliferation and induces apoptosis *TPTEP1* reduces level of *miR-328-5p* in a sponging-dependent manner, upregulates SRCIN1 and influences inactivation of the *Src* and *STAT3* pathways	[141]
*PMPCAP1, SOWAHC*	Chest area	Lung Squamous Cell Cancer	prognostic	Methylation data from TCGA	tissue	*MPCAP1* and *SOWAHC* are hypomethylated higher expressions are associated with poor patient prognosis *PMPCAP1* (as well as *SOWAHC* and *ZNF454*) is involved in gene expression and transcription pathways	[110]
*DUXAP8*	Chest area	Non-Small-Cell Lung Cancer	diagnostic	qRT-PCR of patient samples and cell lines	tissue	it is upregulated in patient tissues and cell lines knockdown of *DUXAP8* represses proliferation, migration, invasion, EMT process and phosphorylation of AKT/mTOR *DUXAP8* has positive correlation with *TRIM44*, while the *miR-498* and *DUXAP8*, as well as *miR-498* and *TRIM44*, are negatively correlated *DUXAP8* regulates the expression of *TRIM44* by *miR-498* knockdown of *DUXAP8* decreases the tumor volume and weight as well as the number of metastatic nodules in vivo	[104,105]
*RPL13AP17, CHIAP2, SFTA1P, SIGLEC17P, CYP2B7P1, CYP4Z2P*	Chest area	Lung Adenocarcinoma	diagnostic and prognostic	RNA-seq (TCGA data),	tissue	*LINC00908, WWC2-AS2* and *CYP2B7P* are independent prognostic risk factors for OS *WWC2-AS2* and *SIGLEC17P* are independent prognostic risk factors for RFS correlation with genes connected with plasma membrane, plasma membrane part, purine nucleotide binding, cytoskeleton, cell adhesion molecules	[101]
*PDIA3P1 (PDIA3P)*	Chest area	Non-Small Cell Lung Cancer	diagnostic and prognostic	RNA-seq (TCGA data) and qRT-PCR of patient samples and cell lines	tissue	it is upregulated in patient samples higher expression is connected with an advanced TNM and lymph node metastasis higher expression is connected with shorter DFS time knockdown of *PDIA3P* suppresses the proliferation and invasion of and reduces tumor growth in vivo *PDIA3P* enhances the activity of the *Wnt/β-catenin* pathway	[109]
*SFTA1P*	Chest area	Lung Squamous Cell Carcinoma	diagnostic and prognostic	RNA-seq (TCGA data) and qRT-PCR of patient samples	tissue	one of the 8 prognosis-associated lncRNAs *SFTA1P* is upregulated in patient samples higher expression is connected with worse survival *MAPK* signaling pathway is associated with *LINC00968, SFTA1P, GATA6-AS1, TBX5-AS1* and *FEZF1-AS1*	[142]
*SUMO1P3*	Chest area	Lung Adenocarcinoma	diagnostic and prognostic	RNA-seq (TCGA data)	tissue	it is upregulated in LUSC and LUAD patient samples it is co-expressed with *SUMO1* higher *SUMO1* or *SUMO1P3* expression is associated with reduced RFS in the case of LUAD patients, but only *SUMO1P3* is the independent prognostic factor	[102]
*SUMO1P3*	Chest area	Non-Small Cell Lung Cancer	diagnostic	RNA-seq (TCGA data) and qRT-PCR of patient samples and cell lines	tissue	it is upregulated in patient sample, cell lines it is correlated with late clinical stage, lymph node metastasis, distant metastasis, and poor differentiated degree *SUMO1P3* has no association with OS and DFS time miR-136 directly binds to *SUMO1P3* and *SUMO1P3* negatively regulates *miR-136,* which regulates the cell phenotype	[103]
*FTH1P3*	Chest area	Non-Small Cell Lung Carcinoma	diagnostic and prognostic	qRT-PCR of patient samples and cell lines	tissue	it is upregulated in patient samples and cell lines higher expression is associated with advanced TNM stage and lymph node metastasis higher expression is associated with poor OS time knockdown of *FTH1P3* suppresses cell migration and invasion in vitro knockdown of *FTH1P3* promotes MET process (decreased expression of N-cadherin, vimentin, and Snail and increased expression of E-cadherin)	[108]
*SLC6A10P*	Chest area	Lung Adenocarcinoma	diagnostic and prognostic	RNA-seq (TCGA data) and ISH of patient samples	tissue	it is upregulated in patient samples higher expression is associated with lymph node metastasis, more advanced tumor stage, and poor overall survival in NSCLC and LUAD patients is an independent prognostic factor for LUAD patients no association with clinicopathological parameters and no prognostic value for LUSC patients	[100]
*CTSLP8, RPS10P20, HLA-K, GPS2P1, LOC387646*	Chest area	Breast Cancer	prognostic	RNA-seq (TCGA) with LASSO-Cox model	tissue	higher expression of *STXBP5*, *GALP* and *LOC387646* indicated poor prognosis for a breast cancer patient increased *CTSLP8* and *RPS10P20* and decreased *HLA-K* pseudogene expression indicates poor prognosis; regarding pseudogene–gene interaction, *GPS2-GPS2P1* improved prognosis, but neither the gene nor pseudogene alone is prognostic of survival *miR-3923* was predicted to target *GPS2* using miRanda, PicTar, and TargetScan, implying modules of gene–pseudogene miRNAs that are potentially functionally related to patient survival	[86]
*HLA-DPB2*	Chest area	Breast Cancer	diagnostic, prognostic and predictive	RNA-seq (TCGA data) and microarray analysis (ONCOMINE)	tissue	pseudogene *HLA-DPB2* and its parental gene *HLA-DPB1* are overexpressed and correlated with better patient prognosis *HLA-DPB2* functions as an endogenous RNA to increase *HLA-DPB1* expression by competitively binding with *miR-370-3p* *HLA-DPB2/HLA-DPB1* axis was strongly connected with immune-related biological functions (associated with high immune infiltration abundance of CD8+ T cells, CD4+ T cells, Tfh, Th1, and NK cells and with high expression of majority biomarkers of monocytes, NK cell, T cell, CD8+ T cell, and Th1 in BC and its subtype), indicating that *HLA-DPB2* influences the abundance of tumor-infiltrating lymphocytes in the microenvironment *HLA-DPB2* and *HLA-DPB1* expression is positively correlated with the expression of *PD-1*, *PDL-1*, and *CTLA-4*	[87]
*RP11-480I12.5-004*	Chest area	Breast Cancer	diagnostic and prognostic	RNA-seq (TCGA data) and qRT-PCR of patient samples and cell lines	tissue	it is upregulated in patient tissue and cell lines knockdown of *RP11-480I12.5* reduces cell proliferation and colony formation, induces cell apoptosis, and inhibits tumor growth in vivo only overexpression of *RP11-480I12.5-004* enhances cell growth in vitro and in vivo *RP11-480I12.5-004* is mainly located in cytoplasm and increases *AKT3* and *CDK6* mRNA expression by competitively binding to *miR-29c-3p* six parental genes of *RP11-480I12.5* are indicated, among which *TUBA1B* and *TUBA1C* are connected with *RP11-480I12.5* expression	[88]
*PCNAP1*	Chest area	Breast Cancer	diagnostic and prognostic	qRT-PCR of patient samples and cell lines	tissue	it is upregulated in patient tissues higher expression is connected with shorter OS time knockdown of *PCNAP1* suppresses the migration and invasion of cells *PCNAP1* functions as a competing endogenous ceRNA for *miR-340-5p* and influences its target *SOX4* and regulates migration and invasion	[89]
*PTENP1*	Chest area	Breast Cancer	diagnostic, prognostic and predictive	qRT-PCR of patient samples and cell lines; databases	tissue	it is downregulated in patient samples and cell lines, especially in advanced and more aggressive forms of cancer higher level is connected with poor clinical prognosis *PTENP1* regulates cell proliferation, invasion, tumorigenesis, and chemoresistance to Adriamycin (ADR) *PTENP1* is a direct target of *miR-20a* and regulates miR expression in sponging-dependent manner and in turn influences *PTEN* expression *PTENP1* activates the *PI3K*/*AKT* pathway and PI3K inhibitor LY294002 or siAKT prevents cancer progression	[97]
*PTTG3P*	Chest area	Breast Cancer	diagnostic and prognostic	RNA-seq (TCGA data), other databases and qRT-PCR of patient samples	tissue	it is upregulated in patient samples is negatively correlated with estrogen receptor (ER) and progesterone receptor (PR) status and positively to basal-like status, triple-negative breast cancer status, Nottingham prognostic index (NPI) and Scarff–Bloom–Richardson grade higher expression is associated with a poor prognosis its expression correlated positively with *PTTG1* expression co-expressed genes with *PTTG3P* are connected with mitotic nuclear division and cell cycle	[91]
*CRYβB2P1*	Chest area	Breast Cancer	diagnostic and predictive	RNA-seq (TCGA data) and qRT-PCR of patient samples and cell lines	tissue	it is upregulated in patient samples depends on patient’s race (increased in African-American relative to White American) *CRYβB2P1* and *CRYβB2* enhance tumorigenesis by promoting cell proliferation *CRYβB2P1* may function as a non-coding RNA regulating *CRYβB2* expression overexpression of *CRYβB2* increases invasive cellular behaviors, tumor growth, IL6 production, immune cell chemoattraction, and the expression of metastasis-associated genes	[92]
*CYP4Z2P*	Chest area	Breast Cancer	diagnostic	qRT-PCR of patient samples and cell lines, RNA-seq and microarray data	tissue	it is upregulated in patient samples it is positively correlated with its parental gene *CYP4Z1* overexpression of *CYP4Z2P*- or *CYP4Z1*-3′UTR activates signaling pathways regulating the pluripotency of stem cells (epithelial cancer stem cells, cell cycle-related genes) overexpression of *CYP4Z1*- or *CYP4Z2P*-3′UTR increases the CD44+/CD24− population *six2* activates the *ceRNET_CC* (*miR-211, miR-125a-3p*, *miR-197*, *miR-1226* and *miR-204*) by binding to their promoters and activates the downstream *PI3K/AKT* and *ERK1/2* pathways the *six2/ceRNET_CC* axis is involved in chemoresistance	[94]
*PDIA3P*	Chest area	Breast Cancer	diagnostic	qRT-PCR of patient samples and cell lines	tissue	it is upregulated in patient samples and cell lines knockdown of *PDIA3P* suppresses cell viability, promotes apoptosis, and inhibits migration and invasion *PDIA3P* is negatively regulates miR-183 and influencing its target *ITGB1*,thus inducing the activation of *FAK/PI3K/AKT/β-catenin* signals and influencing tumor growth and metastasis	[95]
*CKS1BP7*	Chest area	Breast Cancers	diagnostic	Quantitative multi-gene fluorescence in situ hybridization (QM-FISH) technique	tissue	*CKS1BP7* is amplificated in 28.8% of all patients, amplified *IGF1R* in 24.2% amplification of them often co-existed together identical CNAs of *CKS1BP7* and *IGF1R* were found in DCIS and invasive carcinoma within the same tumors amplification of both genes was more frequent in aneuploidy tumors and the tumors with high ki67 no association of amplification and patient outcome	[96]
*FTH1P3*	Chest area	Breast Cancer	diagnostic and predictive	qRT-PCR of patient samples and cell lines	tissue	it is upregulated in paclitaxel-resistant breast cancer tissue and cell lines knockdown of *FTH1P3* decreases the 50% inhibitory concentration value of paclitaxel and induces cell cycle arrest at G2/M phase knockdown of *FTH1P3* suppresses the tumor growth of paclitaxel-resistant breast cancer cells and ABCB1 protein expression in vivo *FTH1P3* promotes ABCB1 protein expression by downregulation of miR-206 in sponging-dependent manner	[98]
*DUXAP8*	Head and neck	Neuroblastoma	diagnostic and prognostic	qRT-PCR of patient samples and cell lines	tissue	it is positively related to the tumor stage it is negatively associated with the patient survival rate knockdown of *DUXAP8* reduces the proliferation, colony formation, cycle, and motility *DUXAP8* regulates *miR-29* expression by sponging-mediated mechanism expression of *NOL4L* is regulated by *DUXAP8*/*miR-29* axis and influence on the cancer progression	[114]
*MT1JP*	Head and neck	Glioma	diagnostic and prognostic	qRT-PCR of patient samples and cell lines	tissue	it is downregulated in patient tissues and cell lines lower expression is associated with glioma progression and poor patient survival overexpression of *MT1JP* reduces the proliferation and invasion *MT1JP* interacts with *miR-24* and negatively regulates its expression level and influences cellular phenotype	[115]
*PDIA3P1*	Head and neck	Glioma	diagnostic and prognostic	Microarray gene profiling dataset GSE45301 and RNA-seq TCGA of patient samples and cell lines	tissue	it is overexpressed and its expression is connected with tumor degree, transcriptome subtype higher level is correlated with poor patient outcomes *PDIA3P1* expression is associated with EMT, disassembly of ECM, and angiogenesis overexpression of *PDIA3P1* enhanced the migration and invasion HIF-1 is confirmed to directly bind to the *PDIA3P1* promoter region and activate its transcription *PDIA3P1* functions as a ceRNA by sponging *miR-124-3p* to modulate *RELA* expression and activate the downstream NF-κB pathway	[116]
*ANXA2P2*	Head and neck	Glioblastoma	diagnostic and prognostic	qRT-PCR of patient samples and cell lines and RNA-seq (TCGA)	tissue	it is upregulated patient tissue and cells knockdown of *ANXA2P2* reduces cell proliferation and aerobic glycolysis and downregulates protein levels of glycolysis markers (GLUT1, HK2, PFK, LDHA) *miR-9* has negative correlation with its own *ANXA2P2* mRNA target overexpression of *miR-9* suppresses the cell proliferation and aerobic glycolysis of glioma cells by bind to *LDHA* 3′UTR	[112]
*RPSAP52*	Head and neck	Glioblastoma	diagnostic and prognostic	qRT-PCR of patient samples and cell lines	tissue	it is upregulated in patient samples higher expression is connected with shorter survival expression level of *RPSAP52* is positively correlated with TGF-β1 overexpression of *RPSAP52* and *TGF-β1* leads to increased and silencing of *RPSAP52* to decreased CD133+ cells (phenotype of cancer initiating cells)	[117]
*PKMP3, AC027612.4, HILS1, RP5-1132H15.3 and HSPB1P1*	Head and neck	Glioma	diagnostic and prognostic	The Cancer Genome Atlas (TCGA) and the Chinese Glioma Genome Atlas (CGGA)	tissue	five pseudogenes (*PKMP3*, *AC027612.4*, *HILS1*, *RP5-1132H15.3* and *HSPB1P1*) are identified as prognostic gene signatures the risk score is an independent prognostic factor pseudogenes are connected with biological processes: *PKMP3* with trans-synaptic signaling, histone modification, *Wnt* and *MAPK* signaling pathways; *AC027612.4* with cell cycle, nuclear division, *PI3K/AKT* and *TP53* signaling pathways; *HILS1* with protein phosphorylation activity and transcriptional misregulation; *RP5-1132H15.3* with microtubule-based movement and ferroptosis; *HSPB1P1* with *JAK* */STAT* cascade, neutrophil mediated immunity, *TNF* signaling pathways and apoptosis upregulation of the genes connected with phagosome, *JAK/STAT, PI3K/AKT*, and *TNF* signaling pathways is observed in high-risk group of patients divided based on five pseudogene signatures	[118]
*ANXA2P2, EEF1A1P9, FER1L4, HILS1, and RAET1K*	Head and neck	Glioma	diagnostic and prognostic	RNA-seq (TCGA data)	tissue	five pseudogenes can used to establish the patient risk signature higher expression of *ANXA2P2*, *FER1L4*, *HILS1*, and *RAET1K*, and lower expression of *EEF1A1P9* are connected with poorer prognosis the risk signature genes are involved in regulation of proliferation, migration, adhesion, ECM receptor interaction, angiogenesis, response to hypoxia (*HIF-1* signaling pathway), *PI3K/AKT* signaling pathway, and apoptosis	[119]
*HERC2P2*	Head and neck	Glioma	diagnostic and prognostic	RNA-seq (TCGA data) and CGGA database of patient samples	tissue	*HERC2P2* is positively correlated with survival it is negatively correlated with clinical grade overexpression of *HERC2P2* reduces migration and colony formation abilities and reduces tumor growth in vivo	[120]
*FTH1P3*	Head and neck	Glioma	diagnostic	qRT-PCR of patient samples and cell lines	tissue	it is upregulated in patient samples and cell lines its expression is higher in high-grade glioma compared with low-grade glioma tissues overexpression of *FTH1P3* promotes glioma cell proliferation and inhibits apoptosis *FTH1P3* inhibits *miR-224-5p* expression, which in turn negatively regulates *TPD52* expression the *FTH1P3/miR-224-5p/TPD52* axis is responsible for glioma progression	[121]
*PTENP1*	Head and neck	Glioma	diagnostic	qRT-PCR of patient samples and cell lines	tissue	it is downregulated in patient samples overexpression of *PTENP1* suppresses cell proliferation (decreases the numbers of S-phase cells) and invasion and migration abilities overexpression of *PTENP1* induces the expression of p21 protein and suppresses the *p38* signaling pathway	[122]
*AGPG*	Head and neck	Esophageal Squamous Cell Carcinoma	prognostic	TCGA analysis and qRT-PCR analysis of patient samples	tissue	highly expressed in many cancers high expression levels are correlated with poor prognosis it is a transcriptional target of *TP53* and loss or mutation of *TP53* induces upregulation of *AGPG* *AGPG* protects *PFKFB3* from proteasomal degradation and leads to the accumulation of *PFKFB3*, which in turn activates glycolytic flux and promotes cell cycle progression knockdown of *AGPG* results in tumor growth in patient-derived xenograft models	[123]
*LILRP1, RP6-191P20.5, RPL29P19, TAS2R2P, and ZBTB45P1*	Head and neck	Head and Neck Squamous Cell Carcinoma	prognostic and predictive	RNA-seq (TCGA data)	tissue	700 differentially-expressed pseudogenes are identified signature of 5 pseudogenes (*LILRP1*, *RP6-191P20.5*, *RPL29P19*, *TAS2R2P*, and *ZBTB45P1*) can distinguish the low-risk and high-risk patients and predicted prognosis with high sensitivity and specificity five pseudogenes are associated with the immune system and cancer-related biological process (*LILRP1* and *RP6-191P20.5* are involved in immune regulation, *PRL29P19* in metabolism regulation, and *TAS2R2P* and *ZBTB45P1* have multiple functions) pseudogene-related pathways enriched in the high-risk group are identified (EMT, angiogenesis, metastasis, proliferation, extracellular matrix receptor, focal adhesion, and *PI3K/AKT* pathways)	[124]
*PTTG3P*	Head and neck	Head and Neck Squamous Cell Carcinomas	diagnostic and prognostic	RNA-seq (TCGA data)	tissue	it is upregulated in patient samples expression depends on the type of mutation in the *TP53* gene, and it correlates with genes from *TP53* pathway expression is correlated with *PTTG1* as well as *PTTG2* expression levels of *PTTG3P* depends on T-stage, grade, and HPV p16 status patients with low expressions of *PTTG3P* have longer DFS time the *PTTG3* high-expressing group of patients has the most deregulated genes connected with DNA repair, oxidative phosphorylation, and peroxisome pathways	[125]
*DUXAP10*	Head and neck	Oral squamous cell carcinoma	diagnostic	Microarray data of GSE30784	tissue	4462 DEGs and 76 differentially expressed lncRNAs were screened between the three groups, and 200 DEGs and only double homeobox A pseudogene 10 (*DUXAP10*) was screened among the three groups 1662 interactions of 46 lncRNAs and their coexpressed target genes were predicted, and 38 pairs of lncRNA-lncRNA coregulated 843 target genes coregulated target genes were significantly enriched in antigen adaptive immune response, activation of phagocytosis receptor signaling, mast granule *NF-κB* inflammation lncRNAs were differentially expressed in OSCC and dysplasia target genes might play an important role in the carcinogenesis and development	[126]
*FKBP9P1*	Head and neck	Head and Neck Squamous Cell Carcinoma	diagnostic and prognostic	qRT-PCR of patient samples and cell lines	tissue	it is upregulated in patient tissues and cell lines higher *FKBP9P1* level is correlated with advanced T-stage, N-stage, and advanced clinical stage higher expression is connected with shorter OS and DFS time knockdown of *FKBP9P1* reduces proliferation, migration, and invasion by reduction of the *PI3K/AKT* signaling pathway activity	[127]
*FTH1P3*	Head and neck	Laryngeal Squamous Cell Carcinoma	diagnostic and prognostic	qRT-PCR of patient samples and cell lines	tissue	it is upregulated in patient samples positively correlated with the poorer differentiation, higher T classification, lymph node metastasis, advanced clinical stage higher *FTH1P3* expression is connected with poorer prognosis overexpression of *FTH1P3* increases cell proliferation, migration, and invasion, and inhibits cell apoptosis	[128]
*TUSC2P*	Head and neck	Esophageal Squamous Cell Carcinoma	diagnostic and prognostic	qRT-PCR of patient samples and cell lines	tissue	it is downregulated in patient samples and cell lines higher expression is associated with better patient survival overexpression of *TUSC2P*-3′UTR results in higher expression of *TUSC2*, inhibition of proliferation and invasion, and promotes apoptosis *TUSC2P*-3′UTR regulates the expression of *miR-17-5p*, *miR-520a-3p, miR-608* and *miR-661* in sponging-dependent manner and protects *TUSC2* mRNA from regulation by these miRNAs	[129,130]
*FTH1P3*	Head and neck	Esophageal Squamous Cell Carcinoma	diagnostic	qRT-PCR of patient samples and cell lines	tissue	it is upregulated in patient samples and cell lines knockdown of *FTH1P3* reduces proliferation, migration, and invasion ability knockdown of *FTH1P3* decreases the expression of *Sp1* and *NF-kB (p65)* and regulates cell phenotype	[143]
*DUXAP10*	Head and neck	Esophageal Squamous Cell Carcinoma	diagnostic and prognostic	qRT-PCR of patient samples and cell lines	tissue	*DUXAP10* was certified to be upregulated in ESCC tissues and cells positively correlated with short survival time down-expression of *DUXAP10* contributed to decreased cell proliferation and metastasis knockdown of *DUXAP10* caused the increased apoptosis rate and stopping of cell cycle *DUXAP10* through recruiting enhancer of zeste homolog 2 (EZH2) to the promoter of p21 influenced on ESCC progression	[144]
*FTH1P3*	Head and neck	Oral Squamous Cell Carcinoma	diagnostic and prognostic	qRT-PCR of patient samples and cell lines	tissue	expression level of *FTH1P3* was significantly upregulated in OSCC tissues and cell lines higher expression of *FTH1P3* was associated with T classification, N classification, and TNM stage low *FTH1P3* expression was associated with better survival *FTH1P3* was an independent prognosis-predicting factor for OSCC patients knockdown of *FTH1P3* reduced the proliferation, migration, and invasion by reduced the activation of *PI3K/AKT/GSK3β/Wnt/β-catenin* signaling	[145]
*DUXAP8*	Head and neck	Oral Cancer	diagnostic and prognostic	RNA-seq (TCGA data) and microarray analysis (GSE30784, GSE74530, GSE84805, GSE125866)	tissue	*DUXAP8* (and other LINC00152, *MIR4435-2HG* and LINC00582) is associated with the patient outcome time knockdown of *DUXAP8* expression reduces cell proliferation through interacting with *EZH2* and repression of *KLF2* expression	[146]

## 4. Conclusions

Even 40 years after the discovery of pseudogenes, knowledge of these genomic components is relatively poor. Hopefully, thanks to the rapid development of the new sequencing technologies, we will be able to identify new pseudogenes and learn more about those already characterized. Silva-Malta et al. recently presented a molecular strategy for the detection of the *RHD* pseudogene (*RHDψ*) based on a real-time polymerase chain reaction (PCR) assay [147]. However, just a certain number of transcriptomes have been covered. Furthermore, while most proposals have led to discovering a targeted algorithm, mainly used for detection, few computational pipelines were designed following a comprehensive approach addressing the identification and quantification of transcriptional activity within a unifying methodological frame. Standard pipelines mainly use the R language and pseudogene databases. Some of them are agnostic, which means that they apply computational tools in a de novo fashion to optimize the detection power, and in turn, to retrieve as many pseudogenes as possible, either annotated or putative ones [148]. Such a four-step pipeline includes (a) mapping RNA-seq samples to the human reference using the spliced-read aligner TopHat; (b) assembling genes and transcripts into putative candidates with Cufflinks [149] and Scripture [150] and comparing them to existing annotations from Ensembl, UCSC, and GENCODE; (c) screening candidate pseudogenes against a collection of features; and (d) appraising putative pseudogenes by using classification algorithms, namely Samtools and Perl.

Although several studies have been performed to date, the extent to which pseudogenes contribute to organismal biology remains largely unclear. The previous obstacles in exploring pseudogenes have been caused by the a priori assumption that they are functionless. Their systematic study has also been hindered by the lack of robust methodologies capable of distinguishing between the biological activities of pseudogenes and the functions of the genes they are derived from. Similarly, lncRNAs were initially dismissed as “junk DNA” or as transcriptional noise, mostly due to their definition as non-protein-coding and generally lower and more restricted expression patterns than mRNAs [131,151]. Future work should seek to explain if pseudogene activation is one of the crucial carcinogenesis factors, or the result of the carcinogenesis process in the situation when no mutation changes in the “driver genes” are observed. [132]. Moreover, some results should be analyzed because some pseudogenes, due to their high similarity to parental genes, give false results, as presented by Zhao et al., who described this problem with the pseudogenes *OCT4pg1*, *OCT4pg3*, and *OCT4pg4* and their parental gene *OCT4* [39]. All of this makes pseudogenes more mysterious than we thought, and they uncover hidden or missed networks of interactions in a cell. We are convinced that through the advancement of technology, genome-wide studies, and detailed biochemical analyses, pseudogenes will be broadly recognized, along with their regulatory potential.

## Data Availability

All data are available online with common access. The analyzed data during the current study are available from the corresponding author on reasonable request.
